# Novel strain of *Pseudoruminococcus massiliensis* possesses traits important in gut adaptation and host-microbe interactions

**DOI:** 10.1080/19490976.2021.2013761

**Published:** 2021-12-29

**Authors:** Kaisa Hiippala, Imran Khan, Aki Ronkainen, Fredrik Boulund, Helena Vähä-Mäkilä, Maiju Suutarinen, Maike Seifert, Lars Engstrand, Reetta Satokari

**Affiliations:** a Human Microbiome Research Program, Faculty of Medicine, University of Helsinki, Helsinki, Finland; b Centre for Translational Microbiome Research, Department of Microbiology Tumor and Cell Biology, Karolinska Institutet, Stockholm, Sweden

**Keywords:** *Pseudoruminococcus*, pili, adhesion, gut microbiota, next-generation probiotic, starch degradation, FMT

## Abstract

Fecal microbiota transplantation (FMT) is an efficient treatment for recurrent *Clostridioides difficile* infection and currently investigated as a treatment for other intestinal and systemic diseases. Better understanding of the species potentially transferred in FMT is needed. We isolated from a healthy fecal donor a novel strain E10-96H of *Pseudoruminococcus massiliensis*, a recently described strictly anaerobic species currently represented only by the type strain. The whole genome sequence of E10-96H had over 98% similarity with the type strain. E10-96H carries 20 glycoside hydrolase encoding genes, degrades starch *in vitro* and thus may contribute to fiber degradation, cross-feeding of other species and butyrate production in the intestinal ecosystem. The strain carries pilus-like structures, harbors pilin genes in its genome and adheres to enterocytes *in vitro* but does not provoke a proinflammatory response. *P. massiliensis* seems to have commensal behavior with the host epithelium, and its role in intestinal ecology should be studied further.

## 1. Introduction

Culture independent detection of microbes in the human gut by high-throughput 16S rRNA gene sequencing and metagenomics has revealed the huge diversity of bacteria associated with this niche. While sequencing technologies allow efficient cataloging of species diversity and the assessment of microbial functions at gene level, cultivation-based methods are essential to characterize bacterial species and their properties *in vitro* and to enable the studying of host-microbe interactions *in vivo*. Thus far, more than one thousand bacterial species associated with the human gut have been cultivated,^1^ but a substantial proportion still remains uncharacterized and uncultured.^2^ A recent metagenomic study discovered almost 2,000 uncultured bacterial species in the human gut and half of the newly discovered species could not be characterized at genus-level, suggesting a vast diversity of unknown bacteria.^2^ During the past decades, sequencing technologies have dominated microbiome research, while culturing of bacteria has received less attention. More recently, however, new culturing efforts have been introduced to obtain pure cultures of bacteria to elucidate their role for human health.^3^

Fecal microbiota transplantation (FMT) is an efficient treatment for recurrent *Clostridioides difficile* infection (rCDI) and actively investigated as a potential treatment for other intestinal and systemic diseases.^4^ In rCDI patients, FMT restores the composition and diversity of disrupted intestinal microbiota,^5,6^ as well as its functionality.^7^ Engraftment of donor microbiota has been associated with favorable treatment outcomes and therefore, the colonization patterns of different species are of special interest as they could be candidates for the development of bacteriotherapy.^6,8^ The potential of different species and strains within the donor´s microbiota to colonize the recipient gut is affected by multiple factors, including the properties of individual strains and co-transferred bacteria as well as the recipient´s endogenous microbiota and niche opportunity in the new ecosystem.^9–12^ The key bacterial species associated with a positive outcome upon FMT are yet to be identified.

Recently, Afouda^13^ et al. isolated a new species, *Pseudoruminococcus massiliensis*, from a healthy Senegalese man, who acted as a fecal donor. The species is strictly anaerobic and belongs to the family Ruminococcaceae. The closest known relative for the species is *Ruminococcus bromii*, which is considered as one of the keystone species within the human gut ecosystem for its ability to degrade resistant starch and its significant contribution to colonic fermentation.^14^ In this study, we isolated from a healthy fecal donor a novel strain of *P. massiliensis*. The strain E10-96H was whole genome sequenced and its properties were studied by using phenotypic tests, transmission electron microscopy (TEM) and *in vitro* functionality tests to assess its interaction with the host epithelium. Our results provide an extended description of *P. massiliensis* to increase our understanding on its role in intestinal microbial ecology and human health.

## 2 Results

### 2.1. Phenotypic characteristics of the fecal isolate *P.* *massiliensis* E10-96 H

*P. massiliensis* E10-96H was isolated from ethanol pre-treated feces of a healthy FMT donor cultivated on yeast casitone fatty acids (YCFA) agar and the species identification was confirmed by whole genome sequencing (WGS, see below). The isolate grew most successfully on fastidious anaerobe agar (FAA) medium as well as in Gifu anaerobic medium (GAM) and reinforced clostridial medium (RCM) broths under anaerobic, but not under aerobic or microaerophilic atmosphere, indicating a strictly anaerobic lifestyle. Cultivation on semisolid GAM agar yielded the strongest growth of the strain. *P. massiliensis* E10-96H appeared in the Gram stain as a Gram-negative diplococcus. Colonies grown on FAA agar were smooth, transparent and 0.5–2 mm in diameter. The strain was non-motile, negative for catalase and oxidase, and the API 20 A, API 20 E, and 20 NE systems did not yield any positive reactions.

### 2.2. Genomic characterization

#### 2.2.1. Genome assembly and genomic features

**Figure 1.**
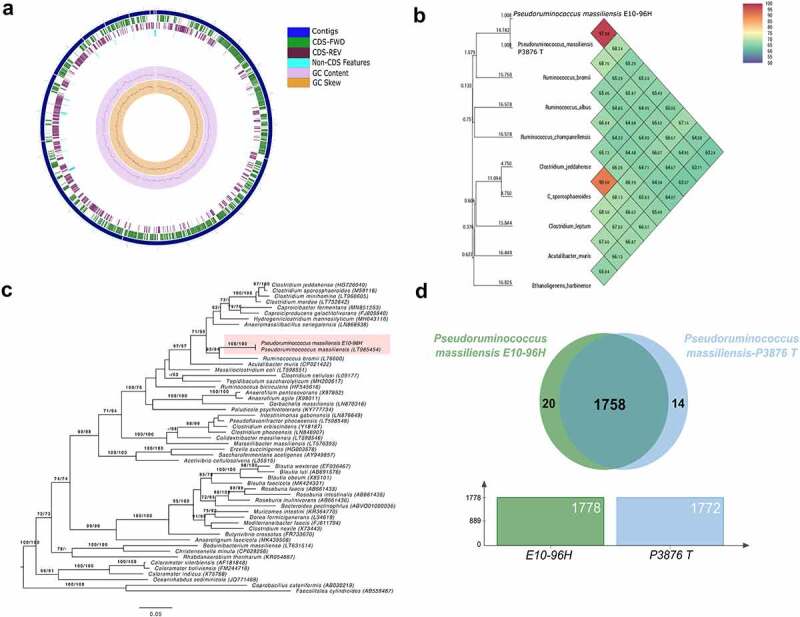
(a) Circular Graphical map of *P. massiliensis* E10-96H genome showing different genomics features. The figures were produced using https://server.gview.ca/. (b) Heatmap showing ORTHO ANI values between *P. massiliensis* E10-96H and other closely related genomes. (c) 16S rRNA gene phylogeny, ML tree inferred under the GTR+CAT model and rooted by midpoint-rooting. The branches are scaled in terms of the expected number of substitutions per site. The numbers above the branches are support values when larger than 60% from ML (left) and MP (right) bootstrapping. (d) The Venn diagram showing shared and unique gene cluster distribution among *P. massiliensis* E10-96H and the type strain of *P. massiliensis.*

The *P. massiliensis* E10-96H genome assembly resulted in 10 contigs. The largest contig was 711,982 bp long, the total size of genome was 2,411,631 bp with N50 value of 617,528 bp, and the GC percentage was 37.1 (Figure 1a). In the genome, 2313 protein coding genes (CDS) and 47 tRNAs (two 5S rRNA, single gene for 16S rRNA and 23S) were found. The annotated genes were characterized for functional categories associated with Clusters of Orthologous Groups (COGs; Table 1).

**Table 1.** Number of genes of *P. massiliensis* E10-96H associated with general Clusters of Orthologous Groups (COGs) functional categories

Class	Count	Coverage	Abundance	Description
J	245	0,52653	0,101268	Translation, ribosomal structure and biogenesis
A	25	0	0	RNA processing and modification
K	231	0,294372	0,090149	Transcription
L	238	0,373949	0,081669	Replication, recombination and repair
B	19	0,052631	0,00045	Chromatin structure and dynamics
D	72	0,277777	0,020816	Cell cycle control, cell division, chromosome partitioning
Y	2	0	0	Nuclear structure
V	46	0,347826	0,035511	Defense mechanisms
T	152	0,289473	0,043497	Signal transduction mechanisms
M	188	0,335106	0,064557	Cell wall/membrane/envelope biogenesis
N	96	0,114583	0,009343	Cell motility
Z	12	0	0	Cytoskeleton
W	1	0	0	Extracellular structures
U	158	0,189873	0,021762	Intracellular trafficking, secretion, and vesicular transport
O	203	0,197044	0,030976	Post-translational modification, protein turnover, chaperones
C	258	0,20155	0,042259	Energy production and conversion
G	230	0,265217	0,067689	Carbohydrate transport and metabolism
E	270	0,411111	0,101089	Amino acid transport and metabolism
F	95	0,48421	0,035741	Nucleotide transport and metabolism
H	179	0,391061	0,054015	Coenzyme transport and metabolism
I	94	0,30851	0,027567	Lipid transport and metabolism
P	212	0,20283	0,042203	Inorganic ion transport and metabolism
Q	88	0,136363	0,012512	Secondary metabolites biosynthesis, transport and catabolism
R	702	0,188034	0,110186	General function prediction only
S	1347	0,098737	0,096852	Function unknown

#### 2.2.2. Species identification and phylogeny

The WGS and 16S rRNA sequence data helped in the species identification and taxonomical assignment resulting in the highest similarity to the type strain *P. massiliensis* Marseille-P3876 T (= CSUR P3876). The ORTHO ANI software^15^ resulted in average nucleotide sequence identity (ANI) of 97.98% between the genomes of the type strain of *P. massiliensis* and isolate E10-96H (Figure 1b). Similarly, 100% sequence identity was observed for 16S rRNA gene sequences of *P. massiliensis* E10-96H and *P. massiliensis* type strain (Figure 1c) and both showed phylogenetic proximity with *R. bromii*. The Type (Strain) Genome Server (TYGS) was also used to cluster species and subspecies. The Genome Blast Distance Phylogeny (GBDP) approach of TYGS also assigned isolate *P. massiliensis* E10-96H to the type strain of *P. massiliensis*. The G + C content variation within species at genome level was less than 1%^16^ (a score of 0.51) which also supported reliable identification.

#### 2.2.3. Analysis of orthologous genes

OrthoVenn2 (https://orthovenn2.bioinfotoolkits.net/home) was used to generate clusters of proteins, orthologs or paralogs, between the type strain of *P. massiliensis* and the isolate *P. massiliensis* E10-96H. An overlapping cluster indicates that the cluster contains proteins shared between the different strains. The type strain of *P. massiliensis* and isolate *P. massiliensis* E10-96H formed 1792 clusters and 1744 single-copy gene clusters. Overall, there were 1758 common clusters shared between the two strains, 20 unique clusters in *P. massiliensis* E10-96H and 14 in the type strain of *P. massiliensis* (Figure 1d).

### 2.3 Starch degradation and genes encoding glycoside hydrolases

When grown on semisolid GAM agar supplemented with potato starch, *P. massiliensis* E10-96H was able to degrade retrograded potato starch creating a clear halo around the bacterial growth, where starch was hydrolyzed (Figure S1). A comparative genomic analysis between *P. massiliensis* E10-96H, *P. massiliensis* type strain and closely related *R. bromii* was performed to reveal the number of the glycoside hydrolases (GHs) present in the two *P. massiliensis* genomes and *R. bromii* ASM283422v1 genome (Table S1). The GH enzymes reportedly range from 50 to 150 in glycan-utilizing human colonic Firmicutes.^14^ In the present study, we found 23 GH enzymes in the *R. bromii* genome compared to the 21 GHs reported earlier^14^ (Table S1). The two new *R. bromii* GHs belonged to GH13 subfamily 28 (Table S2). We found that the only difference between the GH genes in the three genomes was the presence of GH13 subfamily 11 and one extra copy of GH13_42 and GH13_14 in *R. bromii* (Table S2). We also found 20 highly specialized GHs in the *P. massiliensis* E10-96H genome. Out of these 20 members, 14 GHs belonged to family GH13, a hydrolase family dedicated largely to the degradation of starch (Table S1). Similarly to *R. bromii*, out of the 14 GH13 amylases, six GHs also had N-terminal signal peptides and, thus, are possibly secreted (Figure S2). However, we confirmed that unlike *R. bromii*, none of the GH proteins carry a dockerin or cohesin module (Figure S2). The starch-degrading enzymes of *R. bromii* are organized into unique amylosome complexes assembled via interactions between dockerin and cohesin modules.^17^ The remaining six GH enzymes of *P. massiliensis* E10-96H comprised three lysozymes (GH23-GH25), two glucosidases (GH3-GH31) and one amylomaltase (GH77), which specialize in the hydrolysis of α-1,4–linked sugar chains such as amylose. The presence of diverse GHs possibly points toward the nutritional role of the bacterium in starch degradation^14^ (Table S1; Figure S2).

### 2.4 Pilus genes and visualization of cellular protrusions by transmission electron microscopy (TEM)

**Figure 2.**
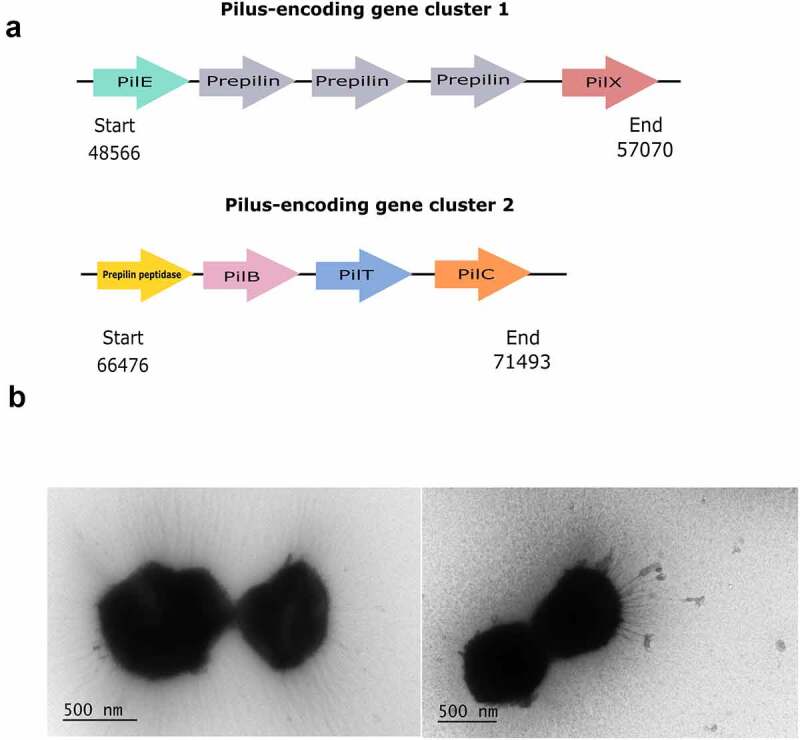
(a) Pilus encoding gene clusters identified in the genome of E10-96H. (b) Transmission Electron Microscopy (TEM) images of bacterium E10-96H showing pilus-like protrusions

In the *P. massiliensis* E10-96H genome, we identified genes involved in type IV fimbrial biogenesis encoding pilin-like proteins (Figure 2a). The type IV pili (T4P) or fimbriae related genes consisted of two pilus-encoding gene clusters. Cluster 1 included pilin subunits PilE and PilX, which are essential for fimbrial biogenesis, natural transformation and protease secretion.^18^ Cluster 2 included prepilin peptidase PilD, PilB, an assembly ATPase involved in the regulation of motility and biofilm formation,^19^ inner membrane protein PilC and PilT, involved in twitching motility and an apparent cytosolic ATPase associated with type IV pilus systems.^18^ PilT is not required for the pilin biogenesis, but is necessary for twitching motility and social gliding behaviors, shown in some bacterial species, powered by pilus retraction.^20^ The T4P undergo extension and retraction processes required for twitching motility with help of two antagonistic cytosolic hexameric ATPases: PilB for polymerization and an inner membrane protein PilC for fimbrial assembly by interacting with PilB.^18^ We also found bifunctional prepilin peptidase PilD in the PilB and PilC gene cluster of *P. massiliensis* E10-96H genome (Figure 2a). PilD is needed for cleavage of the leader peptide and for methylation of the new N-terminal phenylalanine residue of the pilin subunit prior to its assembly into filamentous fimbriae. In addition to the major pilin subunit, several pilin-like proteins that contain an overall positively charged leader peptide preceding a conserved hydrophobic N-terminal domain are involved in type IV fimbrial biogenesis and its associated functions, such as natural transformability and epithelial cell adherence.^18^ Overall, our findings suggest that *P. massiliensis* E10-96H possibly belongs to the T4aP model with biogenesis machinery typically harboring a PilT retraction ATPase.^21^

When *P. massiliensis* E10-96H was grown on semi-solid GAM agar, the bacterium formed cellular protrusions visible by TEM (Figure 2b). The filamentous structures of the bacterium, clearly visible in multiple different TEM images, indicated the presence of pili or fimbriae. This supports our findings of *P. massiliensis* E10-96H harboring genes encoding pilin-like proteins.

### 2.5. *In vitro* interaction with the intestinal epithelium

**Figure 3.**
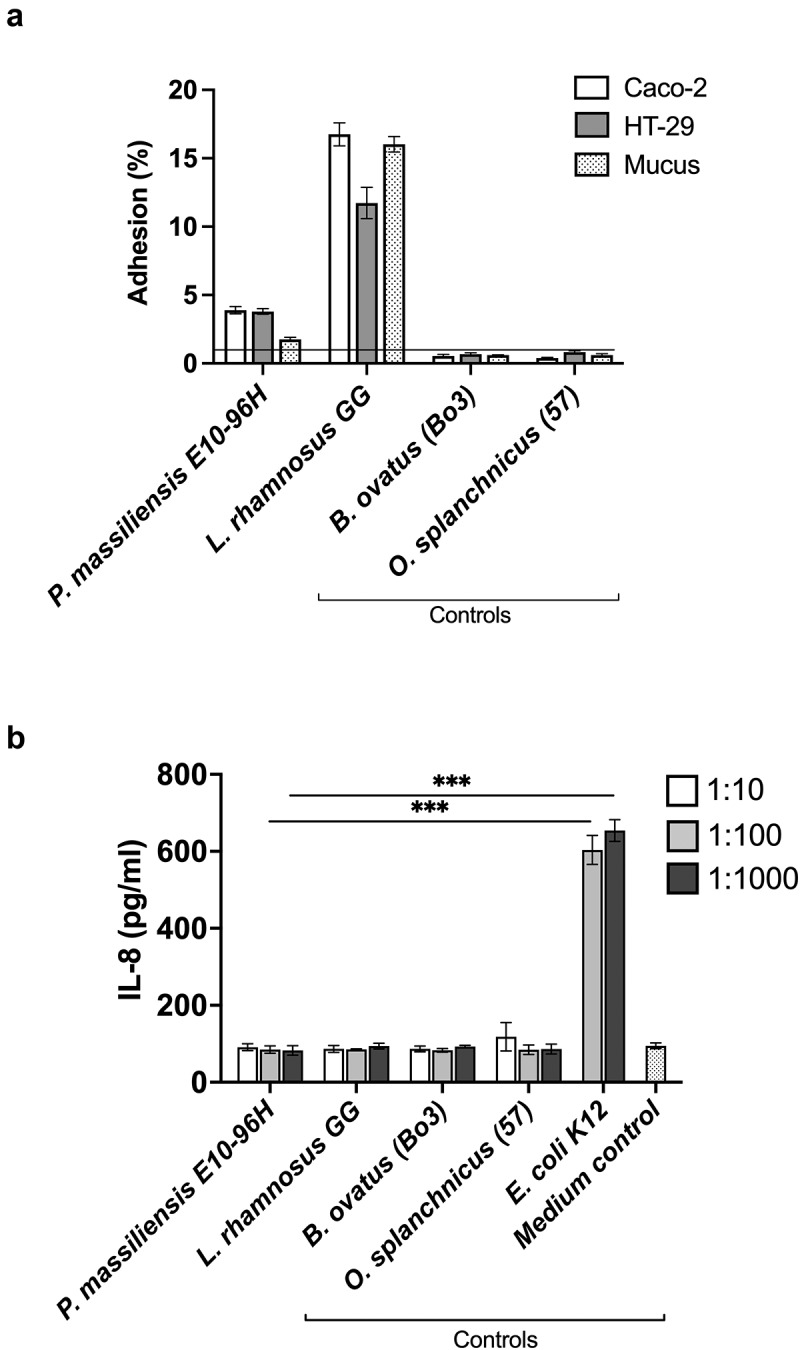
(a) Adhesion of *P. massiliensis* isolate E10-96H to intestinal epithelial cell lines and mucus. Data is shown as means of adhered bacteria (% of total added bacteria) and standard deviations of four technical replicates (parallel wells) from a representative experiment. *L. rhamnosus* GG was used as a positive control. *O. splanchnicus* 57^23^ and *B. ovatus* Bo3^24^ were used as negative controls. Line represents threshold of 1% which is considered as nonspecific binding. B) IL-8 release from HT-29 cells induced by 1:10, 1:100, and 1:1000 dilutions from OD_600 nm_ 0.25 adjusted cell suspensions of the isolate *P. massiliensis* E10-96H, *E. coli* K12, *L. rhamnosus* GG, *O. splanchnicus* 57^23^ and *B. ovatus* Bo3^24^. The 1:10 dilution is not shown for *E. coli* due to excess toxicity. Growth medium for HT-29 cells was used as a control. Results from a representative experiment are shown as means and standard deviations of three replicates (parallel wells). *** = *p* < .001

Next, we tested the adhesion capacity of *P. massiliensis* E10-96H to enterocytes (Caco-2 and HT-29 cell lines) and mucus (Figure 3a). *Lacticaseibacillus rhamnosus* strain GG (ATCC53103; LGG), known to adhere exceptionally well,^22^ was used as a positive control, whereas fecal bacterial isolates *Odoribacter splanchnicus* 57^23^ and *Bacteroides ovatus* Bo3^24^ were chosen as non-binding, negative controls. The isolate E10-96H could bind to both cell lines (relative adhesion level approximately 4%), whereas the binding to mucus was less efficient (1.8%). The adherence of isolate E10-96H to enterocytes was found to be at the level that is typical for intestinal isolates based on our previous research,^23,24^ albeit it was three-times lower compared to the positive control LGG, which is known for its strong adherence.^22^

We also studied the potential proinflammatory effect of *P. massiliensis* E10-96H on enterocytes and found that it did not evoke the release of IL-8 cytokine in HT-29 cell line (Figure 3b). *E. coli* K12, used as a positive control, showed expected proinflammatory effect and induced a strong IL-8 response in HT-29 cells. LGG, *B. ovatus* Bo3^24^ and *O. splanchnicus* 57^23^, included as non-inducing controls, did not promote IL-8 release from HT-29 cells as the cytokine levels were at similar level compared to the medium control, alike in the case of *P. massiliensis* E10-96H. Furthermore, we also measured the production of two other proinflammatory cytokines, IL-1β and TNF-α, in HT-29 cells after the incubation with *P. massiliensis* E10-96H, *E. coli* K12, LGG, *B. ovatus* Bo3 and *O. splanchnicus* 57. However, the levels of IL-1β and TNF-α were very low and could not be reliably quantified. The result is in line with previous observations that epithelial cells, such as HT-29 cells, produce IL-8 but only minor amounts of other proinflammatory cytokines.^25,26^

## 3. Discussion

The human intestinal isolate E10-96H is the second strain representing the recently described genus *Pseudoruminococcus* and our study provides an amended description of *P. massiliensis*, which is the only species of the genus.^13^ We discovered that the gut isolate *P. massiliensis* E10-96H harbors pilus-like structures, can adhere *in vitro* to intestinal epithelium, but does not induce a proinflammatory response in enterocytes and belongs to the group of starch degrading colonic commensals, similarly to its closest phylogenetic relative, *R. bromii*.

Like the previously published type strain of *P. massiliensis*,^13^ the strain E10-96H is a Gram-negative diplococcus, non-motile, catalase and oxidase negative and an obligate anaerobe. The API systems 20 A, 20 E, and 20 NE did not yield any information about the strain’s metabolism. Although these systems are designed for the identification of specific anaerobes (API 20 A), Enterobacteriaceae and other non-fastidious Gram-negative rods (API 20 E), and non-fastidious, non-enteric Gram-negative rods (API 20 NE), they also provide a convenient method for assessing metabolism of bacterial strains. However, the API systems yielded comprehensively negative results for E10-96H, which may indicate the strain’s inability to survive in the systems without the presence of some essential nutrients and vitamins rather than its inability to metabolize any of the substrates. Indeed, by using semisolid GAM agar as the growth medium, we were able to show that E10-96H is able to degrade retrograded potato starch i.e., resistant starch. The phylogenetic tree revealed the evolutionary proximity of our strain E10-96H with *R. bromii*, a dominant member of the human gut microbiota having a key role in starch degradation,^14,17^ which prompted us to compare the GH gene repertoire in the genomes of the two species, *P. massiliensis* and *R. bromii*. Comparative genomics revealed an overall similar GH gene family repertoire in the two species, with most of the genes encoding for hydrolases belonging to the GH13 family. Taken together, our results indicate that *P. massiliensis* E10-96H is able to hydrolyze resistant starch.

The ability to degrade complex carbohydrates is an important trait of a specific subset of bacteria in the complex gut ecosystem.^27^ The first degraders of insoluble polysaccharides, such as *R. bromii, Eubacterium rectale* and *Bifidobacterium* spp.^27^ break down non-digestible food particles in the colon releasing energy sources for the microbial community as part of bacterial cross-feeding system. The bacterial capacity to ferment resistant starch has an important function in cross-feeding species that produce butyrate, which is a key bacterial metabolite regulating gut homeostasis for example by enhancing the epithelial barrier function and ameliorating inflammation.^28^

Intriguingly, we found *P. massiliensis* E10-96H to harbor T4P genes and produce extracellular protrusions visualized by TEM. Bacterial pili reside on the surface of the cell and are comprised of a conserved structural subunit that generally has an N-terminal methylated residue, often phenylalanine, a conserved hydrophobic N-terminal domain, and a C-terminal disulfide bond.^18,29^ Initially bacterial pili most likely facilitated motility and/or DNA uptake.^30^ The evolutionary diversification of the T4P or type IV filament (TFF) superfamily, involving multiple gene duplications, gene fissions and deletions, and accretion of novel components, helped in functional innovation and diversification to flagellar or twitching motility, gliding motility, adhesion, biofilm, protein secretion, and DNA uptake.^30^ In the human gastrointestinal environment, pili or fimbriae are essential for bacterial adhesion to mucus, epithelial cells and food components enhancing the survival and persistence of the bacteria in the gut.^21^ In the genome of *P. massiliensis* E10-96H, we identified two pilus-encoding gene clusters, which comprise most of the genes known to be required for type IV fimbrial biogenesis. The presence of extracellular hair-like appendages can play an important role in the successful colonization of *P. massiliensis* E10-96H upon FMT. Our *in vitro* adhesion results showed that *P. massiliensis* E10-96H binds to Caco-2 and HT-29 enterocyte cell lines at the same level as several other intestinal species such as *Bacteroides, Sutterella* and *Akkermansia* spp.^24,31,32^ However, the adhesion of *P. massiliensis* E10-96H to mucus was much lower, and thus it may not adhere strongly to the colonic epithelium, which is covered by a thick mucus layer protecting the epithelial cell layer. It is possible that *P. massiliensis* E10-96H pilus-like structures facilitate other functions in the colonic environment and also in the gut lumen, such as binding to food particles, especially complex carbohydrates, or formation of biofilms, as described for *Ruminococcus albus*^33^ and *Lactococcus lactis*.^34^

In conclusion, an anaerobic intestinal isolate E10-96H from a healthy fecal donor was identified as *P. massiliensis* based on the WGS data and our studies showed that *P. massiliensis* possesses traits that may have importance in its adaptation to the gut environment. Firstly, *P. massiliensis* E10-96H carries pilus-like structures which may mediate adhesion to intestinal epithelium or food particles, and be utilized in colonization and nutrient harvesting, respectively. Secondly, *P. massiliensis* E10-96H is able to degrade resistant starch and based on its almost similar GH gene repertoire as the known keystone resistant starch hydrolyzing bacterium *R. bromii*, it may play a pivotal role degrading complex carbohydrates and cross-feeding other species in the gut. Furthermore, *P. massiliensis* E10-96H does not provoke proinflammatory responses in enterocytes suggesting a commensal role with the host epithelium. This study extends the recent description of new species *P. massiliensis* and provides first insights into its interaction with the human host. The role of *P. massiliensis* in the complex gut microbial ecosystem should be studied further and the isolated strains open multiple exciting paths for further studies of the putative beneficial interactions of *P. massiliensis* with the human host.

## 4. Materials and methods

### 4.1. Isolation and identification

E10-96H was isolated from the feces of a healthy, pre-screened fecal donor. The use of the donor sample was approved by the Ethics Committee of Hospital District of Helsinki and Uusimaa Finland (DnroHUS124/13/03/01/11). The donor provided a written informed consent.^6^ The frozen fecal solution (saline-10% glycerol) was thawed anaerobically and serially diluted in PBS. The sample was treated with 70% ethanol (1:1) for four hours under aerobic conditions and cultivated on reduced YCFA agar plates in an anaerobic chamber (Whitley MG500 Anaerobic Workstation). After 96 hours of anaerobic incubation, colonies were picked, re-streaked on new agar plates and purified. 16S rRNA gene sequencing was used for the tentative identification of the isolate.

### 4.2. Phenotypic characterization

The strain’s ability to grow under different environmental conditions was examined by inoculating the strain on solid GAM (Nissui Pharmaceutical) and solid Difco™ RCM (BD), and growing the cultures at 37°C for 48 ± 4 h under aerobic, anaerobic (85% N2, 10% CO_2_, and 5% H2; Whitley A85 workstation, Don Whitley Scientific) and microaerophilic (5% O_2_, 10% CO_2_, and 85% N_2_; jar with a CampyGen™ sachet, Oxoid™) atmosphere.

The strain was Gram stained for Gram reaction as well as cell morphology and tested for catalase and oxidase activity. Motility was examined by inoculating the strain into semisolid GAM (0.75% w/v agar) and then observing the growth patterns of the stab culture after 48 h incubation under anaerobic atmosphere at 37°C. The strain’s metabolic capabilities and substrate utilization was tested by API 20 E, API 20 A and API 20 NE systems (bioMérieux). Testing was done according to the manufacturer’s instructions except that incubation of systems took place under anaerobiosis and the incubation temperature for API 20 NE was 37°C instead of 30°C. The strain’s ability to hydrolyze retrograded starch was examined using amylase test by growing the strain on semisolid GAM supplemented with 0.5% (w/v) of potato starch. After incubation in conditions described above, the medium was stained with potassium iodide to observe starch hydrolysis.

### 4.3. Genomic DNA isolation, library preparation, and whole genome sequencing

Bacterial genomic DNA was isolated using MagAttract HMW DNA extraction kit (Qiagen) according to the manufacturer’s instructions, except Metapolyzyme enzyme mix (Sigma) was used as an alternative to lysozyme. DNA was eluted to EB buffer and the concentration was measured using a NanoDrop spectrophotometer. The library preparation was done using Rubicon ThruPLEX DNA-seq Kit (Takara) with 350 bp. Clustering was done by ‘cBot’ and samples were sequenced on NovaSeq6000 (NovaSeq Control Software 1.4.0/RTA v3.3.3) with a 2 × 151 setup using ‘NovaSeqXp’ workflow in ‘S2ʹ mode flowcell. The Bcl to FastQ conversion was performed using bcl2fastq_v2.19.1.403 from the CASAVA software suite. The quality scale used was Sanger/phred33/Illumina 1.8 + .

### 4.4. WGS data analyses

The paired end fastq reads were processed using the BACTpipe v.2.7.0 where mash screen^35^ was used to confirm the purity of reads belonging to single species, bbduk^36^ performed reads quality trimming and filtering, and FastQC was used for quality evaluation. De-novo genome assembly was done by Shovill (https://github.com/tseemann/shovill) using SPAdes^37^ at its core and finally, genome annotation was done using prokka and RAST algorithm.^38,39^

We used EzBioCloud: database of 16S rRNA and whole genome assemblies^40^ together with TYGS^41^ for species identification and phylogenetic inference. For genome-scale taxonomic analysis, the genome assemblies were searched using BBMap’s MinHash Sketch^35^ to first identify the closely related type strain genomes. EzBioCloud: database of 16S rRNA and whole genome assemblies^40^ was also used for identification of similar closely related species to bacterium genome. OrthoANI measures the overall similarity between two genome sequences.^15^ ANI and OrthoANI are comparable algorithms: they share the same species demarcation cutoff at 95 ~ 96% and large comparison studies have demonstrated both algorithms to produce near identical reciprocal similarities. TYGS clusters species and subspecies using the dedicated clustering algorithm and established thresholds^42^ analogous to 70% and ca. 79% DDH, respectively. Further 16S rRNA gene phylogenies were also inferred using the DSMZ phylogenomics pipeline at http://ggdc.dsmz.de/. A multiple sequence alignment was created with MUSCLE.^43^ Maximum likelihood (ML) and maximum parsimony (MP) trees were inferred from the alignment with RAxML^44^ and TNT,^45^ respectively. For ML, rapid bootstrapping in conjunction with the autoMRE bootstopping criterion^46^ and subsequent search for the best tree was used; for MP, 1000 bootstrapping replicates were used in conjunction with tree-bisection-and-reconnection branch swapping and ten random sequence addition replicates. The sequences were checked for a compositional bias using the Χ^2^ test as implemented in PAUP*.^47^

The genome level comparison and annotation of orthologous gene clusters was carried out using OrthoVenn2 (https://orthovenn2.bioinfotoolkits.net/home).

### 4.5. Pilus and starch degradation genes

To accurately identify putative pilins, we used different approaches, Blast^48^ and Hmmer^49^ together with PilFind algorithm which makes use of type III signal sequence motif (http://signalfind.org /pilfind.html).^50^

The genome-wide distribution of genes encoding carbohydrate-active enzymes (CAZys) was performed specifically for GHs. BLASTp was used to search closely related GH13 protein sequences. The protein sequences were assigned to carbohydrate-active enzymes (CAZymes) as per CAZy database (http://www.cazy.org).^51,52^ The profile hidden Markov model (HMM) libraries were used by HMMER software suite (http://hmmer.org) to predict the Pfam domains.^49^

### 4.6. Other bacterial strains and growth conditions

*E. coli* K12-derived TOP10 (Invitrogen, USA) was cultivated overnight in Luria–Bertani broth (Becton Dickinson, USA) under aerobic conditions at 37°C. LGG (ATCC53103), *O. splanchnicus* 57 and *B. ovatus* Bo3 were grown in GAM broth anaerobically at 37°C for 48 hours.

### 4.7. Epithelial cell lines

The human colonic epithelial cell lines Caco-2 (ACC169) and HT-29 (ACC299) were purchased from the German Collection of Microorganisms and Cell Cultures (DSMZ). Both cell lines were grown at 37°C in an incubator under an oxic atmosphere with 5% CO_2_ and passaged every 3–4 days after reaching 70–80% confluence using TryplExpress (Lonza, USA) to detach the cells. HT-29 cells were cultivated in McCoy 5A (Lonza, Belgium) medium containing 10% heat-inactivated (30 min at 56°C) fetal bovine serum (FBS; Gibco) and 100 U ml-1 PEST. Caco-2 cells were grown in RPMI 1640 medium (Sigma-Aldrich, USA) supplemented with 20% FBS, nonessential amino acids (1%, NEAA; Lonza, Belgium), 15 mM HEPES (Lonza, Belgium), 100 U ml-1 penicillin and streptomycin (PEST; Lonza, Belgium) and 2 mM L-glutamine (Lonza, Belgium). Passages 6–28 were used in the experiments.

### 4.8. Sample preparation and TEM

Bacterial sample was diluted 1:2 and prepared for electron microscopy by loading to carbon coated and glow discharged 200 mesh copper grids with pioloform support membrane.^53^ Sample was fixed with 2.0% PFA in NaPO4 buffer, stained with 2% neutral uranyl acetate, further stained and embedded in uranyl acetate and methyl cellulose mixture (1.8/0.4%). Bacterial cells were viewed with transmission EM using Jeol JEM-1400 (Jeol Ltd., Tokyo, Japan) operating at 80 kV. Images were taken with Gatan Orius SC 1000B CCD-camera (Gatan Inc., USA) with 4008 × 2672 px image size and no binning.

### 4.9. Adhesion

The adherence of *P. massiliensis* E10-96H to Caco-2 and HT-29 cell lines (8 days after plating) and mucus was studied as previously described.^24^ Shortly, *P. massiliensis* E10-96H, positive control LGG and negative controls *O. splanchnicus* 57 and *B. ovatus* Bo3 were grown in GAM medium supplemented with 10 μl ml-1 of [6ʹ-3H]thymidine (17,6 Ci mmol-1, Perkin Elmer, USA), which metabolically radiolabels the bacterial cells. Four technical replicates (parallel wells) were used in each experiment. Porcine mucus (Sigma-Aldrich, 50 μg per well in PBS) was allowed to absorb to Maxisorp microtiter plate wells overnight at 4°C. 10,000 Caco-2 or HT-29 cells per well were seeded onto 96-well microplates. [3H]Thymidine-labeled bacterial cells were washed with an appropriate medium (McCoy 5A for HT-29 cells, RPMI 1640 for Caco-2 cells and PBS for the mucus assay) and adjusted to OD_600nm_ 0.25 which corresponds approximately 10^8^ cells/ml. After one hour of incubation on the epithelial cell monolayer or mucus at 37°C, the bacterial suspensions were removed, and the wells were washed three times to remove the non-adherent bacteria. The adhered bacteria were lysed with 1% SDS-0.1 M NaOH solution overnight at 37°C. Radioactivity was measured with a liquid scintillator (Wallac Winspectral 1414, Perkin-Elmer, Waltham, MA, USA). The adhesion percentage was calculated relative to the radioactivity of the bacterial suspension initially added to the wells.

### 4.10. Proinflammatory cytokine induction in HT-29 cells

Induction of proinflammatory cytokine IL-8, IL-1β and TNF-α response in HT-29 cells (8 days post-plating) by *P. massiliensis* E10-96H was carried out as previously described.^23^ In brief, the bacterial suspension was washed with McCoy 5A medium supplemented with 10% FBS and adjusted to OD_600nm_ 0.25. Bacterial dilutions of 1:10, 1:100, and 1:1,000 were used in the experiment. The diluted suspensions were incubated on the HT-29 cells for 3 h at 37°C in a CO_2_ incubator. *E. coli* K12 was used as a proinflammatory control. Three technical replicates (parallel wells) were used in each experiment. An OptEIA Human IL-8 ELISA kit (BD Biosciences, USA), human TNF-alpha DuoSet (R&D systems, Biotechne, USA) and human IL-1beta/IL-1F2 DuoSet (R&D systems, Biotechne, USA) were used according to the manufacturer’s instructions to measure the concentration of the cytokine in the culture media. A two-sample t-test was used to determine significant differences between *P. massiliensis* E10-96H and *E. coli* K12. The analysis was carried out using GraphPad Prism 8.4.1 (GraphPad Software, United States). A *p*-value of <0.05 was considered statistically significant.

## Supplementary Material

Supplemental Material

## Data Availability

The data that support the findings of this study are openly available in European Nucleotide Archive at https://www.ebi.ac.uk/ena/browser/home, reference number PRJEB46251.
