# Supplementary material for: Novel strain of Pseudoruminococcus massiliensis possesses traits important in gut adaptation and host-microbe interactions
Source: Gut Microbes. 2021 Dec 29;14(1):2013761. doi: 10.1080/19490976.2021.2013761 (PMC8726730; doi:10.1080/19490976.2021.2013761)
Supplement: Supplemental Material [file KGMI_A_2013761_SM5905.zip › supplementary/downloadFromZipFile.pdf]

Novel strain of Pseudoruminococcus massiliensis possesses traits important in gut adaptation and host-microbe interactions

SUPPLEMENTARY MATERIAL

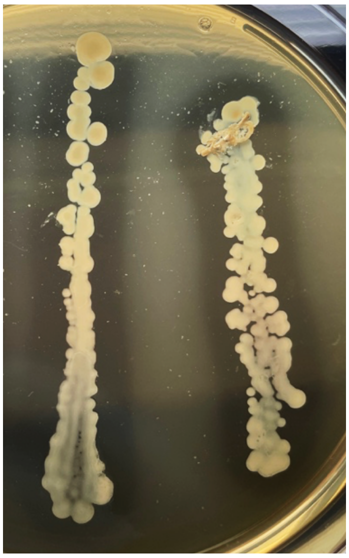

Figure S1. A clear halo around the growth of *P. massiliensis* on semisolid GAM agar supplemented with potato flour after addition of 10% iodine solution.

Table S1. Glycoside hydrolase (GH) gene families in the *P. massiliensis* E10-96H genome compared to *P. massiliensis* type strain Marseille-P3876 T genome and *R. bromii* reference genome ASM283422v1

**Species**	**Glycoside Hydrolase Family**							
	**GH3**	**GH13**	**GH23**	**GH24**	**GH25**	**GH31**	**GH77**	**Total GH**
***P. massiliensis* E10-96H**	**1**	**14**	**1**	**1**	**1**	**1**	**1**	**20**
***P. massiliensis* Marseille-P3876 T**	**1**	**14**	**1**	**1**	**0**	**1**	**1**	**19**
***R. bromii* ASM283422v1**	**1**	**17**	**1**	**1**	**1**	**1**	**1**	**23**
***R. bromii* (Ze et al. 2015)**	**1**	**15**	**2**	**0**	**1**	**1**	**1**	**21**

Table S2. Glycoside hydrolase gene family 13 of *P. massiliensis* E10-96H compared to *P. massiliensis* type strain Marseille-P3876 T and *R. bromii* ASM283422v1

**Species**	**Glycoside Hydrolase Family 13 Genes**										
	**9**	**11**	**Unassigned**	**14**	**19**	**20**	**28**	**36**	**39**	**42**	**Total**
***P. massiliensis* E10-96H**	2	0	2	2	1	1	3	1	1	1	14
***P. massiliensis* Marseille-P3876 T**	2	0	2	2	1	1	3	1	1	1	14
***R. bromii* ASM283422v1**	2	1	2	3	1	1	3	1	1	2	17
***R. bromii* (Ze et al. 2015)**	2	1	2	3	1	1	1	1	1	2	15

GH13_42 | E10_96H_00151 Beta/alpha-amylase

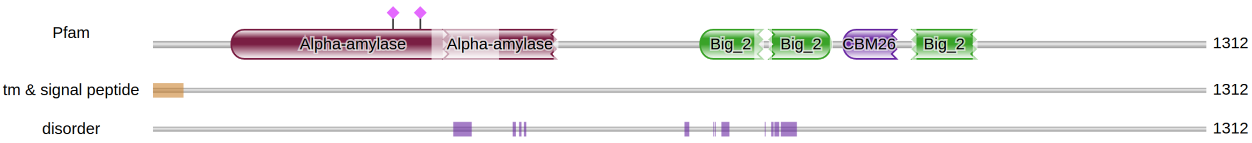

GH13_39 | E10_96H_01773 Amylopullulanase

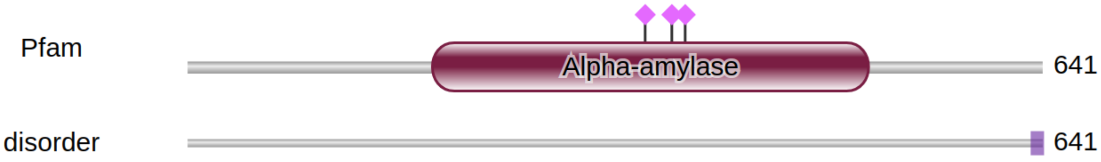

GH13_19 | E10_96H_00277 Periplasmic alpha-amylase

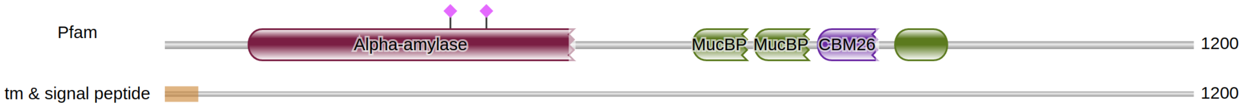

GH13_36 | E10_96H_00858 Alpha-amylase

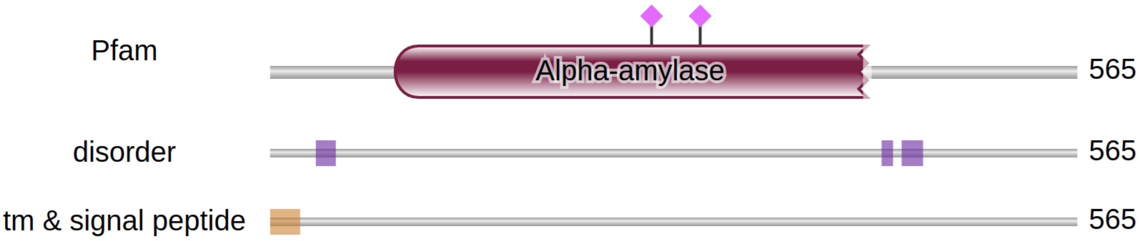

GH13 | E10_96H_00637 Alpha-1,4-glucan, maltose-1-phosphate

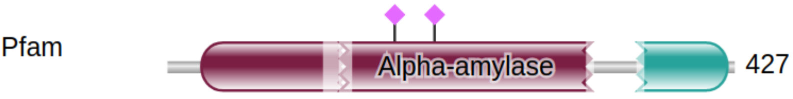

GH13 | E10_96H_00168 Trehalose synthase/amylase TreS

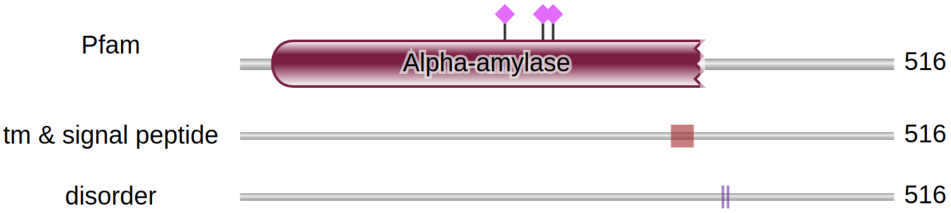

GH13_20 | E10_96H_01014 Neopullulanase

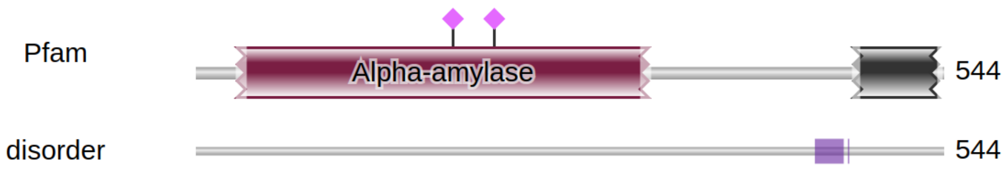

GH13_28 | E10_96H_00150 Alpha-amylase

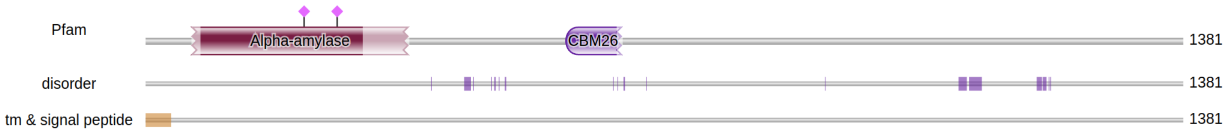

GH13_28 | E10_96H_00909 Alpha-amylase

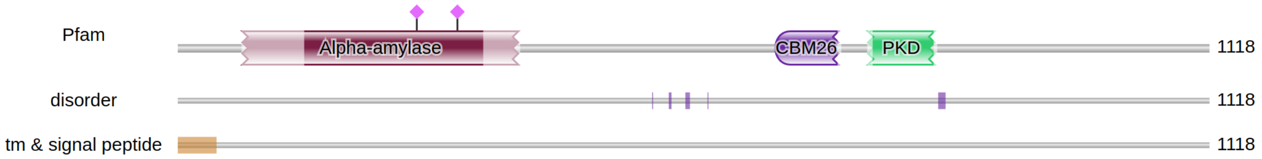

GH13_28 | E10_96H_02083 Alpha-amylase

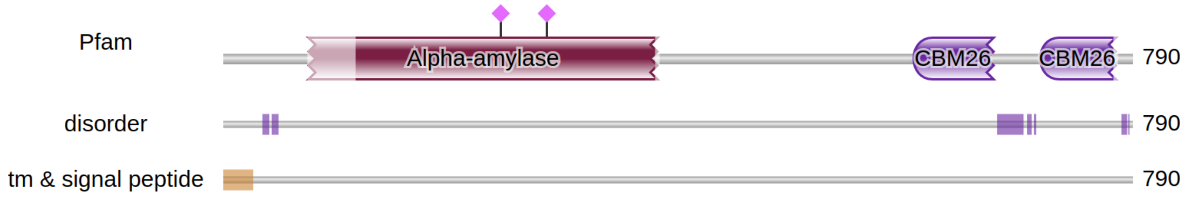

GH13_14 | E10_96H_01793 Pullulanase

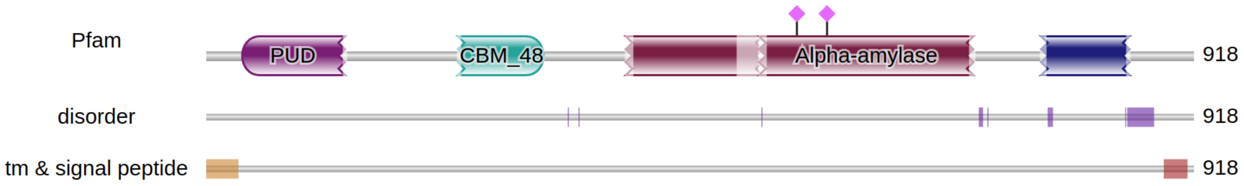

GH13_14 | E10_96H_00013 Pullulanase

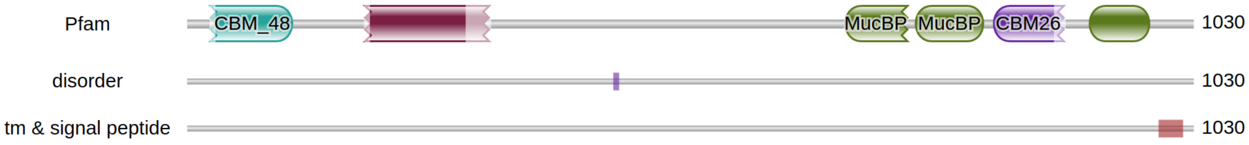

GH13_9 | E10_96H_00610 1,4-alpha-glucan branching enzyme GlgB

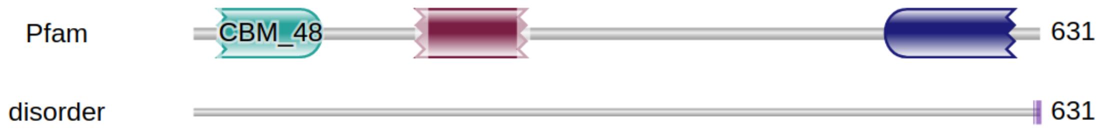

GH13_9 | E10_96H_00886 1,4-alpha-glucan branching enzyme GlgB

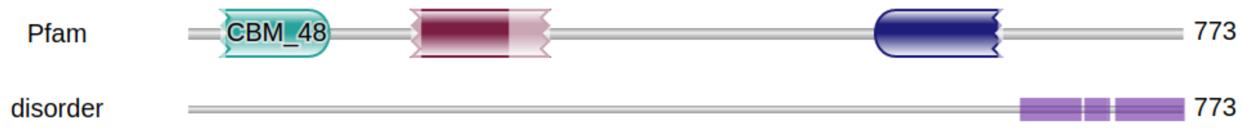

Figure S2. Modular organization of glycoside hydrolase (GH) 13 catalytic modules, alpha amylases and signal peptides in the genome of *P. massiliensis* isolate E10-96H
